# Inhibitor of Apoptosis Proteins: Promising Targets for Cancer Therapy

**DOI:** 10.4172/2157-2518.S14-004

**Published:** 2013-05-27

**Authors:** Thomas W Owens, Andrew P Gilmore, Charles H Streuli, Fiona M Foster

**Affiliations:** 1 Wellcome Trust Centre for Cell Matrix Research, Faculty of Life Sciences, University of Manchester, Manchester, M13 9PT, UK; 2 Department of Physiology, Sydney Medical School & Bosch Institute, the University of Sydney, NSW, Australia

**Keywords:** IAP, Apoptosis, Cytokines, Extracellular matrix, Cancer therapy, Clinical trials

## Abstract

Cancer is a disease in which normal physiological processes are imbalanced, leading to tumour formation, metastasis and eventually death. Recent biological advances have led to the advent of targeted therapies to complement traditional chemotherapy and radiotherapy. However, a major problem still facing modern medicine is resistance to therapies, whether targeted or traditional. Therefore, to increase the survival rates of cancer patients, it is critical that we continue to identify molecular targets for therapeutic intervention. The Inhibitor of Apoptosis (IAP) proteins act downstream of a broad range of stimuli, such as cytokines and extracellular matrix interactions, to regulate cell survival, proliferation and migration. These processes are dysregulated during tumourigenesis and are critical to the metastatic spread of the disease. IAPs are commonly upregulated in cancer and have therefore become the focus of much research as both biomarkers and therapeutic targets. Here we discuss the roles that IAPs may play in cancer, and the potential benefits and pitfalls that targeting IAPs could have in the clinic.

## Introduction

Since their discovery almost 20 years ago, the Inhibitor of Apoptosis (IAP) family of proteins have gathered growing interest as possible drug targets in a wide range of malignancies. IAPs are commonly upregulated in cancer, and although initially thought to only regulate cell death, they are now known to be involved in many aspects of both normal tissue function and tumour development. In this review we will focus on summarising how IAPs affect the signalling pathways dysregulated in cancer and the current IAP-based therapies that are in development.

**Figure 1:**
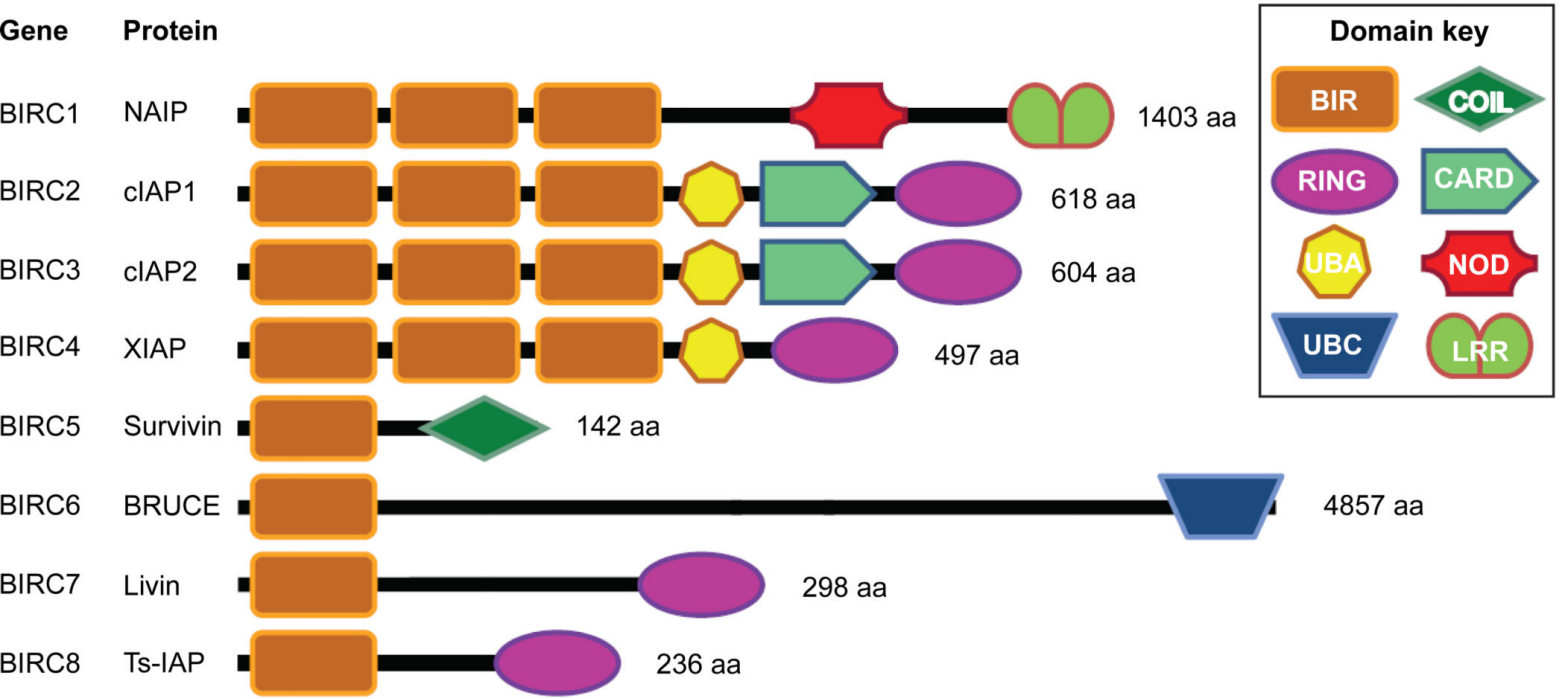
Schematic representation of human IAPs IAPs contain between one and three Baculovirus IAP repeat (BIR) domains, a 70–80 amino acid Zinc-binding motif. Five of the 8 IAPs possess a carboxy-terminal RING (really interesting new gene) domain that functions as an E3 ligase, capable of self-ubiquitination and ubiquitination of associated proteins. BRUCE lacks a RING domain but possesses an Ubiquitin-Conjugating Domain (UBC) that can induce ubiquitination. XIAP and cIAPs have an Ubiquitin-Associated (UBA) ubiquitin-binding domain that is important for their signalling function [67,98]. In addition cIAP1 and cIAP2 contain a Caspase Recruitment Domain (CARD) that can mediate homotypic interactions [99]. NAIP possesses a LRR (Leucine-Rich Repeat) and a NOD (nucleotide-binding oligomerisation domain), which have been implicated in microbial pathogen recognition. Survivin contains a COIL (coil-coiled) domain, which is involved in binding to chromosomal paasenger proteins INCENP and borealin.

The IAPs were first discovered in baculoviruses, where they were found to encode for proteins (cpIAP, OpIAP) able to inhibit apoptosis in the host cell [1,2]. IAPs are evolutionarily conserved and defined by the presence of at least 1 Baculovirus IAP Repeat (BIR) domain. In humans there are 8 IAPs (genes birc1–8), NAIP, cIAP1, cIAP2, XIAP, Survivin, BRUCE/Apollon, Livin and Ts-IAP (Figure 1). In addition to the BIR domains, IAPs possess a number of other distinct functional domains that impart broader functionality on mammalian IAPs than their viral counterparts [3–5].

## Core Functions of IAPs

From early over-expression studies, it was proposed that IAPs prolong cell survival by inhibiting the activity of initiator (caspase-9) and effector (caspases-3&−7) caspases by binding to the active caspases [6]. However, XIAP is now known to be the only mammalian IAP that is a bona fide caspase inhibitor [7]. XIAP also ubiquitinates caspases via its E3 ubiquitin ligase domain, resulting in caspase degradation or inactivation [8–10].

**Figure 2:**
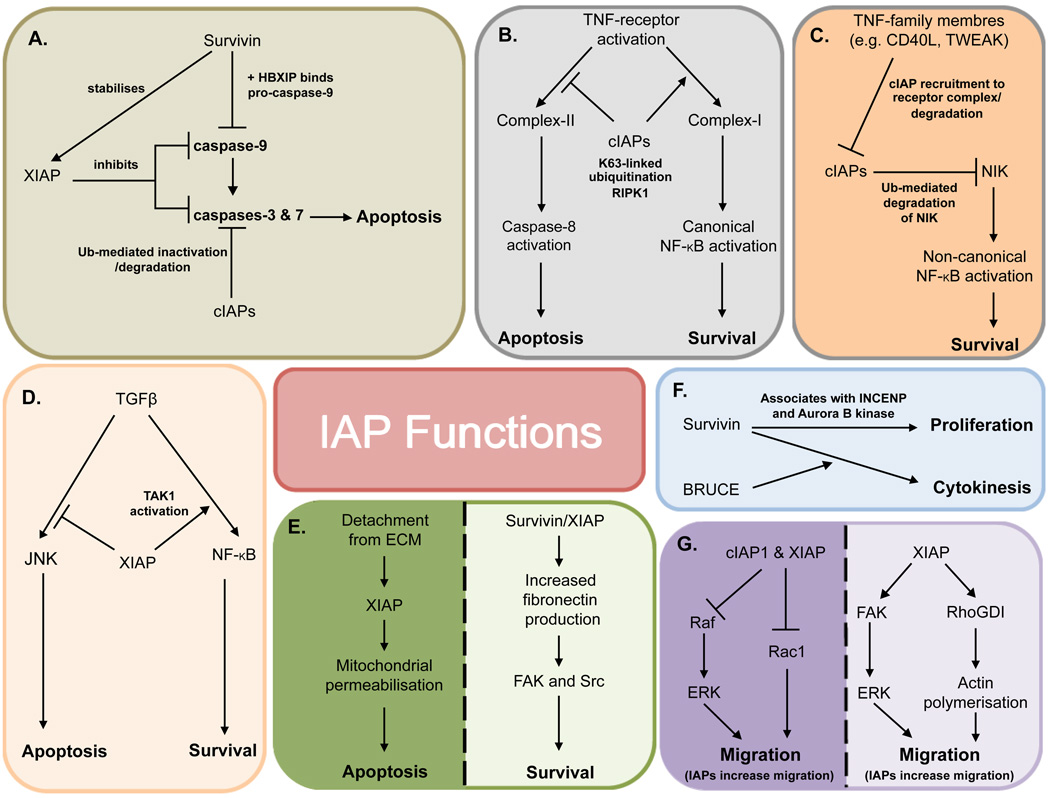
Summary of IAP functions A selection of the pathways in which IAPs function to regulate apoptosis, survival, cell cycle and migration: **A** –Regulation of caspases, **B** – TNFα signalling, **C** – Non-canonical NF-κB**D** – TGFβ signalling, **E** –ECM interactions, **F** – Cell cycle, **G** – Migration.

Survivin, in the presence of HBXIP co-factor, binds to and inhibits pro-caspase 9, preventing its recruitment to Apaf1 [11]. In addition Survivin interacts with XIAP, resulting in stabilisation and synergistic inhibition of caspase 9 [12]. The cIAPs, while being able to bind to caspases, do not directly inhibit caspase activity and instead they mediate caspase ubiquitination and degradation [13,14] (Figure 2A).

It is now known that caspase regulation represents only a small proportion of the mechanisms by which IAPs impact cell longevity. IAPs also regulate cytokine signals and have a role in linking cell-ECM interactions to survival. Moreover, IAPs are signalling effectors in a range of additional cellular processes, including cell cycle and migration (Figure 2B–G).

### The role of IAPs in survival signalling

Tumour necrosis factor α (TNFα) is a pleiotropic cytokine, associated with the generation of an inflammatory response. Following TNFα binding to TNF-R1, both TRADD and RIP1 are rapidly recruited to the receptor complex. TRADD then recruits TRAF2, which associates with cIAP1 and 2 to form the survival-inducing “Complex-I”. Polyubiquitination of RIP1 in a non-degradative Lys63 manner by cIAP1 and cIAP2 allows the recruitment of proteins that activate canonical NF-κB signalling, leading to upregulation of survival proteins, such as c-FLIP [15]. In the absence of cIAPs, NF-κB is not activated and the failure to upregulate c-FLIP leads to TNF-induced activation of caspase-8 and apoptosis via formation of a death-inducing “Complex-II” [16,17] (Figure 2B).

As well as influencing the canonical NF-κB pathway, cIAPs affect the non-canonical NF-κB pathway through regulation of NIK [18]. In unstimulated cells, ubiquitin-mediated degradation of NIK by cIAPs prevents non-canonical NF-κB activation. Following activation of receptors belonging to the TNFR superfamily, such as CD40, cIAPs are recruited to the receptor complex, freeing NIK to accumulate and activate non-canonical NF-κB signalling [19] (Figure 2C). Therefore, cIAPs have significant roles in multiple NF-κB pathways.

Downstream of TGFβ receptor activation, NF-κB is activated by 2 main pathways. In the first pathway, XIAP induces transcription of NF-κB responsive genes in a Smad4 dependent manner [20]. In the second pathway XIAP forms a complex with TGFβ-activated kinase 1 (TAK1) and its binding partners TAB1 and TAB2, resulting in TAK1 activation. TAK1 phosphorylates the NF-κB inhibitor, IκB, resulting in its proteasomal degradation and the activation of NF-κB. TGFβ mediated TAK1 activation results in the upregulation of the pro-apoptotic p38 and JNK signalling pathways [21]. However, NF-κB activation induces transcriptional upregulation of XIAP, which then mediates ubiquitination and proteasomal degradation of TAK1 to suppress pro-apoptotic JNK signalling in a pro-survival feedback loop [5,22] (Figure 2D).

IAPs also regulate other pro-survival signalling cascades via their E3 ligase domain. For example, XIAP ubiquitinates PTEN leading to Akt phosphorylation and activation [23]. cIAP1 ubiquitinates the c-myc regulatory protein, MAD1, thereby activating c-myc. In this context, cIAP1 acts synergistically with c-Myc to enhance tumour formation [24]. cIAPs can also promote MAPK-dependent cell proliferation and survival in TNF-stimulated cells. The cIAPs ubiquitinate TRAF3 (TNFR associated factor 3), thereby allowing TRAF2/6:MAPK translocation to the cytosol and activation [25].

### The role of IAPs in ECM-mediated survival

Interactions between cells and their surrounding Extracellular Matrix (ECM) mediate the spatial control of cell fate, and are crucial in determining the survival of normal cells [26,27]. Perturbation of ECM-adhesion signals induces apoptosis by activating Bax-driven mitochondrial permeabilisation [28]. Survivin and XIAP cooperate to upregulate several ECM proteins, particularly fibronectin [29]. The resulting fibronectin-activated signalling via FAK and Src kinases promotes survival in response to altered adhesion of cells to ECM [29]. Interestingly, as an early response to altered cell-ECM interactions, XIAP can translocate to mitochondria where it forms a 400 kDa complex and contributes to mitochondrial permeabilisation [30]. Therefore, depending on temporal context, IAPs can both protect cells or promote ECM-regulated apoptosis (Figure 2E).

### The role of IAPs in cell cycle

Although cytoplasmic Survivin has a role in promoting cell survival, its primary function is in the nucleus, where it is required along with Aurora B kinase and INCENP to form the chromosomal passenger complex during mitosis. Survivin knockout embryos die at E4.5 due to failed cytokinesis while cells lacking Survivin display a multiploidy phenotype [31,32]. Interestingly, the survival and proliferative functions of Survivin are spatially distinct. Survivin contains a nuclear export sequence and can be found in both the nucleus and the cytosol: while nuclear Survivin regulates proliferation, the cytoplasmic protein acts to suppress apoptosis [33,34]. Moreover, Survivin, along with its family member Bruce, are also implicated, in cytokinesis [35] (Figure 2F).

### The role of IAPs in migration

IAPs have a number of roles in cell migration. XIAP is recruited via caveolin-1, to α5-integrin adhesion complexes, where it interacts with focal adhesion kinase (FAK). This α5-integrin:caveolin:FAK complex is required for activation of ERK-dependent shear stress–induced endothelial cell migration [36–38]. Furthermore, XIAP can promote migration and invasion via interactions with RhoGDI and subsequent regulation of actin polymerisation [39].

In contrast to promoting migration, IAPs can also inhibit cellular motion. XIAP and the cIAPs bind to c-Raf, resulting in ubiquitination of c-Raf, in a manner dependent upon the Hsp90-mediated quality control system, but independent of their E3-ligase activity. Knockdown of XIAP resulted in stabilisation of c-Raf and increased c-Raf-dependent cell migration [40]. Additionally, XIAP and cIAP1 can reduce migration by mediating the proteasomal degradation of Rac1 [41]. Therefore, IAPs influence cell migration in a context-dependent manner (Figure 2G).

## IAPs contribute to cancer progression

The above discussion reveals that IAPs have a broad portfolio of roles in regulating cell survival, proliferation and migration. Moreover, IAPs are regulated during normal developmental programmes that become subverted in cancer [42]. It is therefore not surprising that there is an ever-expanding body of evidence connecting changes in IAP expression with tumourigenesis [43–46].

### Survivin

The survivin gene is among the top 5 cancer-associated genes. It is upregulated in the vast majority of cancers and is associated with resistance to both chemo and radio-therapy, as well as a poor prognosis [47]. The divergent functions of nuclear and cytoplasmic Survivin are highlighted in studies on breast cancer where elevated levels of cytoplasmic Survivin correlate with a poorer patient outcome owing to its anti-apoptotic function, while increased nuclear Survivin is correlated with a better outcome [31,33,48–51].

As a chromosomal passenger protein, Survivin acts to stabilise microtubules, which results in resistance to chemotherapeutics, such as Vinca alkaloids [52]. Survivin, via its interaction with Aurora B kinase, may also function to promote the indefinite proliferation potential of cancer cells by upregulating human telomerase reverse transcriptase [53].

Perhaps surprisingly, Survivin also regulates cancer cell autophagy by interacting with the key autophagy regulator, Beclin [54]. In glioma cells, knockdown of Beclin resulted in decreased Survivin levels, and increased apoptotic sensitivity to TRAIL. In prostate cancer cells, chemokine-mediated protection from autophagic cell death is mediated by upregulation of Survivin [55].

### XIAP

XIAP expression is upregulated in a variety of cancers, including breast, lung, renal and bladder carcinoma [56–59]. XIAP may mediate anoikis resistance to contribute to tumour metastasis [60,61]. However, the correlation of XIAP expression and prognosis is unclear. Increased XIAP levels correlate with disease severity in acute myeloid leukaemia and prostate cancer, but not non-small cell lung carcinoma [58,62,63]. In a recent study in which XIAP was stably over-expressed at levels 2–5 times higher than normal, which is similar to levels seen in cancer samples, XIAP only provided chemoresistance when combined with the loss of the XIAP antagonist, Smac/DIABLO. Therefore, elevated levels of XIAP alone may not be a prognostic indicator [64].

### cIAPs

Genomic changes in cIAPs are associated with some tumour types. For example chromosomal amplification of 11q21–q23, which encodes both cIAP1 and cIAP2, has been observed in oesophageal squamous cell carcinomas [65,66]. In MALT (mucosal associated lymphoid tissue) B cell lymphomas, cIAP2 gene translocation results in expression of a cIAP2-MALT fusion protein. This drives constitutive NF-κB activation, via a UBA domain dependent binding of NEMO [67]. Similarly, the UBA domain of cIAP1 has also been shown to be essential for cIAP1-mediated oncogenesis [67]. cIAP1 and cIAP2 are often over-expressed in cancers along with YAP, as they are all located within the same genetic locus. In fact, in hepatoma, cIAP and YAP cooperate to induce tumourigenesis [68,69].

## IAPs are potential therapeutic targets in the clinical setting

The overwhelming data that IAPs suppress apoptosis, enhance survival signalling and are upregulated in many cancer types argues that they may be excellent therapeutic targets. Particularly appealing is the possibility that IAP antagonists might specifically target cancer cells over normal cells.

Numerous pre-clinical studies have shown that targeting IAPs, with either siRNAs or mimetics of the naturally occurring IAP antagonist Smac/DIABLO, increases sensitivity of cancer cells to therapies that are widely used in the clinic (Table 1). As a consequence IAP drug development has progressed at a rapid pace, such that multiple IAP inhibitors have been developed and some of these have progressed to in- patient clinical trials (Table 2). Discussed below is a selection of the therapeutics and different approaches used to target IAPs in cancer.

### Antisense based therapies

An antisense oligonucleotide directed against XIAP (AEG35156) is in phase I/II clinical trials for patients with pancreatic, breast, non-small cell lung cancer, AML, lymphoma and solid tumours in which docetaxel is the drug of choice. Although AEG35156 in its ‘first-in-man’ study was well tolerated, it had little significant effect on patient outcome in pancreatic ductal adenocarcinoma or acute myeloid leukaemia [70,71]. Studies involving patients with non-small cell lung carcinoma were terminated due to unacceptable neurotoxicity in two of the patients.

### Smac mimetics

Another method of targeting IAP function is using “Smac mimetics”, which are molecules developed based on the IAP-Binding Motif (IBM) of the potent IAP-antagonist, Smac (also known as DIABLO). Several Smac mimetics are currently in pre-clinical or phase I trials (Table 2). These inhibitors were initially developed as a means of inhibiting XIAP, but it has since been shown that the Smac mimetics also induce the degradation of cIAPs [72]. The loss of cIAPs means that following ligand engagement of the TNF-receptor, Complex I matures into Complex II, leading to caspase-dependent apoptosis [16,17]. Ligand-independent Smac mimetic-induced cIAP degradation causes Ripoptosome (a FADD-caspase 8, RIP1 complex) formation, leading to death via apoptosis or necroptosis [15,73].

At high doses, Smac mimetics induce death in a subset of cancer cell lines in a caspase 8, and TNFα dependent mechanism, but do not induce apoptosis in non-malignant cells [74–77]. Perhaps more importantly, Smac mimetics can work synergistically with other treatments. They sensitise pancreatic cancer cells and glioblastoma cells to γ-irradiation, and breast cancer cells to etoposide, Herceptin, and TRAIL [59,78].

### Other small molecule inhibitors

Several small molecule inhibitors directed against Survivin have been developed. YM155, an inhibitor designed to suppress Survivin promoter activity, showed promise in phase I trials, induced stable disease in 9 / 33 patients in one study and significant tumour shrinkage and remission in another phase I study [79,80]. Phase II trials with YM155 showed favourable results in refractory non small cell lung carcinoma and B cell lymphoma but not in melanoma [81–83]. YM155 is also effective in pancreatic cancer cell culture and xenograft models [84].

Hsp90 stabilises Survivin, and targeting Hsp90 can result in proteasomal degradation of Survivin followed by mitochondrial-mediated apoptosis. Therefore drugs that target Hsp90 may also influence Survivin levels and patient outcome. Such drugs include Shepherdin, and AICAR, which are respectively in pre-clinical and phase II clinical trials.

### Immune-based therapies

Sera from breast, lung and GI cancer patients contain antibodies to Survivin, suggesting that anti-cancer vaccines may be generated [85]. Recently, a phase I trial in which 9 patients with urothelial cancer were vaccinated against Survivin showed no adverse side effects. Five of the 9 patients had an increase in Survivin peptide specific cytotoxic T cells and one patient showed decreased tumour volume [86]. In a second study, a Survivin minigene DNA vaccine induced a 48 – 52% reduction in tumour volume, weight and metastasis in a syngeneic neuroblastoma mouse model. Therapeutic vaccination of the syngeneic neuroblastoma mice led to eradication of neuroblastoma in 50% of the mice and decreased tumour growth by 80% in the remaining mice [87].

Overall, from the available data on pre-clinical trials and initial ‘in-man’ trials, IAP based therapies may indeed be beneficial in the fight against cancer. Significantly though, targeting IAPs may also help to overcome resistance of cancer to existing therapeutics.

## IAPs contribute to the acquired resistance of cancer therapies

IAP levels can increase following the onset of drug treatment. This may provide a mechanism for therapeutic resistance. For example, cisplatin treatment of prostate cancer cells resulted in upregulation of Survivin, XIAP and cIAP2; adriamycin-resistant MCF7 breast cancer cells showed upregulation of XIAP and Survivin; and Lapatinib-resistant BT474 breast cancer cells had elevated Survivin levels. Moreover, survival of adriamycin resistant HL-60 cells were dependent on upregulation of XIAP and MRP (multidrug resistant protein) [88–91]. Importantly, these increases in IAP levels might contribute to acquired drug resistance, one of the major hurdles facing clinicians today. Cancer cells using IAPs as a method to escape chemotherapy highlights another reason why targeting IAPs may be useful to combating the disease.

## Notes of caution to targeting IAPs

The majority of current research supports the idea that IAPs are promising therapeutic targets in cancer, but a few notes of caution remain. Therapies targeting Survivin reduce clonogenic survival of cancer cells and increase rates of apoptosis, usually downstream of mitotic catastrophe. However, loss of Survivin can result in the generation of polyploidy cells, which are more susceptible to the accumulation of mutations and genetic instability. Therefore, cancer cells that escape anti-Survivin based therapies may form a more aggressive transformed phenotype than observed in the original cancer [92]. Moreover, although Survivin is not expressed in differentiated adult human cells it is still expressed in adult proliferating cells, such as the cells of the immune system [93]. Thus, as with other chemotherapies, the effect of Survivin antagonists on the immune system would need to be carefully monitored, especially in the megakaryocyte and haempoietic populations.

Therapies aimed at inhibiting XIAP may result in increased rates of apoptosis in sensitised type II cells, such as hepatocytes. In response to death receptor activation, type II cells require the activation of Bid to amplify the apoptosis signal and commit cells to death. Removal of XIAP from this system also removes the requirement for Bid and results in greater rates of apoptosis. Therefore, combining XIAP antagonism with therapies that activate death receptors may result in high liver toxicity in patients [94]. As XIAP can promote the ubiquitination and degradation of c-RAF, XIAP-targeted therapies could also increase cell migration via c-RAF stabilisation and activation of the MAPK signalling cascade [40].

In contrast to many cancers where IAP upregulation occurs, the biallelic deletion of cIAP1 and cIAP2 is associated with a poorer prognosis in multiple myeloma [95,96]. Despite the expectation that cells lacking the cIAPs would be more sensitive to TNFα, this is not the case in multiple myeloma. It is therefore possible that using Smac mimetics in certain specific situations may enhance carcinogenesis. The ability of Smac mimetics to activate NF-κB signalling will also require careful attention. Treatment of mice with the Smac mimetics stimulated osteoclastogenesis and induced osteoporosis by inducing NIK-dependent activation of NF-κB [97]. Bone loss caused by Smac mimetics may be counteracted by administration of bisphosphonates, such as zoledronic acid [97].

## Conclusion

IAPs are much more than just “inhibitors of apoptosis”. An involvement with signal transduction cascades regulating apoptosis, proliferation, cell survival and migration strongly implicates IAPs with cancer progression and a growing body of work supports the concept of targeting IAPs to treat cancer. Combining anti-IAP therapies with traditional drug approaches has tremendous promise for the future care of cancer patients.

## Figures and Tables

**Table 1:** Pre-clinical data where IAP inhibition sensitised to anti-cancer therapies.

IAP	Cancer	Mechanism of inhibition	Increased sensitivity to	Ref.
**XIAP**	Colorectal	shRNA	TRAIL Taxanes γ-irradiation	[100]
	Breast	shRNA	TRAIL Taxanes	[101]
		siRNA	Etoposide Doxorubicin	[102]
			Lapatinib	[59]
	Lung	Antisense	Doxorubicin Taxol Vinorelbine Etoposide	[103]
		siRNA	Cisplatin	[68]
	Pancreatic	siRNA	Doxorubicin Paclitaxol	[104]
	Melanoma		Dacarbazine TRAIL	[105]
**Survivin**	Lung	siRNA	Adriamycin	[106]
			Cisplatin Paclitaxol	[107]
	Breast	siRNA	Adriamycin	[108]
	Melanoma		TRAIL	[105]
	Hepatocellular	siRNA	Radiotherapy	[109]
		Antisense	TRAIL	[110]
**cIAP2**	Pancreatic	siRNA	Doxorubicin Paclitaxel	[104]
	Oral Squamous	siRNA	5-flurouracil	[111]
	Colorectal			[112,113]
**cIAPs**	Glioblastoma	Smac mimetic	Imatinib	[114]
		Smac mimetic (BV6)	γ-irradiation	[78]
	Non small cell lung carcinoma	Smac mimetic (JP1201)	Doxorubicin Erlotinib Gemcitabine Paclitaxol Vinorelbine	[115]
		Smac mimetc (BV6)	Radiotherapy	[116]
	Breast	Smac mimetic (SM164)	TRAIL	[116]
		Smac mimetic (Compound C)	Herceptin	[59]
	Prostate	Smac mimetic (SM164)	TRAIL	[117]
	Colon			

**Table 2:** IAP-based therapies in clinical trials.

IAP	Drug	Company	Mode of action	Clinical Trial	Ref.
**XIAP**	AEG35156	Aegera Therapeutics	Antisense	Phase 1	[118]
	Embelin		Small molecule targeting BIR3 domain	Pre-clinical	[119,120]
	Polyphenylureas / Xantags	Burnham Institute	Small molecule targeting BIR2 domain	Pre-clinical	[121]
	Arylsulfonamides (TWX006, TWX024)	Novartis		Pre-clinical	[122]
**Survivin**	LY2181308	Eli Lily	Antisense	Phase 2	[92,93]
	YM155	Astellas Pharma Inc	Small molecule antagonist	Phase 2	[123]
	Shepherdin		Small molecule targeting Hsp90	Pre-clinical	[124,125]
	AICAR			Phase II	[126]
	Anti-Survivin Ab		Antibody	Phase 1	[85]
**cIAPs and XIAP**	TL32711 (Birinapint) Compound A	TetraLogic Pharma	Smac mimetic	Phase 1 /2	[76]
	AEG40826 (HGS1029),	Aegera Therapeutics		Phase 1	[17]
	AEG40730	Aegera Therapeutics		Phase 2	
	Compound 8, BV6, SM-122, SM-164	Ascenta Therapeutics		Pre-clinical	[75]
	AT-406	Ascenta Therapeutics		Phase 1	[127]
	Compound 3	University of Texas / Joyant		Phase 1	[74]
	LBW242, LCL-161	Novartis		Phase 1	[128]
	Compound C	Genentech		Phase 1	[129]
	Compound 11	Pfizer		Pre-clinical	[130]
	JP-1201			Pre-clinical	[115]

## Abbreviations

BIR: Baculovirus IAP Repeat
BRUCE: BIR Containing Ubiquitin-Conjugating Enzyme
c-FLIP: Cellular FLICE Inhibitory Protein
CARD: Caspase Recruitment Domain
cIAP: Cellular IAP
ECM: Extracellular Matrix
HBXIP: Hepatitis B X-Interacting Protein
Hsp90: Heat Shock Protein 90
IAP: Inhibitor of Apoptosis
IκB: Inhibitor of Kappa B
INCENP: Inner Centromere Protein
JNK: cJun N-Terminal Kinase
MAD1: MAX Dimerisation Protein 1
MAPK: Mitogen Activated Protein Kinase
MAX: Myc Associated Factor X
NAIP: Neuronal Apoptosis Inhibitory Protein
NEMO: NF-κB Essential Modulator
NF-κB: Nuclear Factor Kappa B
NIK: NF-κB-Inducing Kinase
PTEN: Phosphatase and Tensin Homolog
RIP: Receptor-Interacting Protein
Smac/DIABLO: Second Mitochondrial Activator of Caspases/Direct IAP-Binding Protein With Low pI
TAB1: TAK1 Binding Protein
TAK1: TGF Beta-Activated Kinase 1
TGFβ: Transforming Growth Factor Beta
TNF: Tumour Necrosis Factor
TNF-R: TNF Receptor
TRADD: Tumor Necrosis Factor Receptor Type 1-Associated DEATH Domain
TRAF: TNF Receptor Associated Factor
TRAIL: TNF-Related Apoptosis-Inducing Ligand
Ts-IAP: Testis Specific IAP
UBA: Ubiquitin-Associated
UBC: Ubiquitin-Binding Domain
XIAP: X-Linked IAP
